# Supplementary figures and images for: DNA methylation in human gastric epithelial cells defines regional identity without restricting lineage plasticity
Source: Clin Epigenetics. 2022 Dec 30;14:193. doi: 10.1186/s13148-022-01406-4 (PMC9801550; doi:10.1186/s13148-022-01406-4)

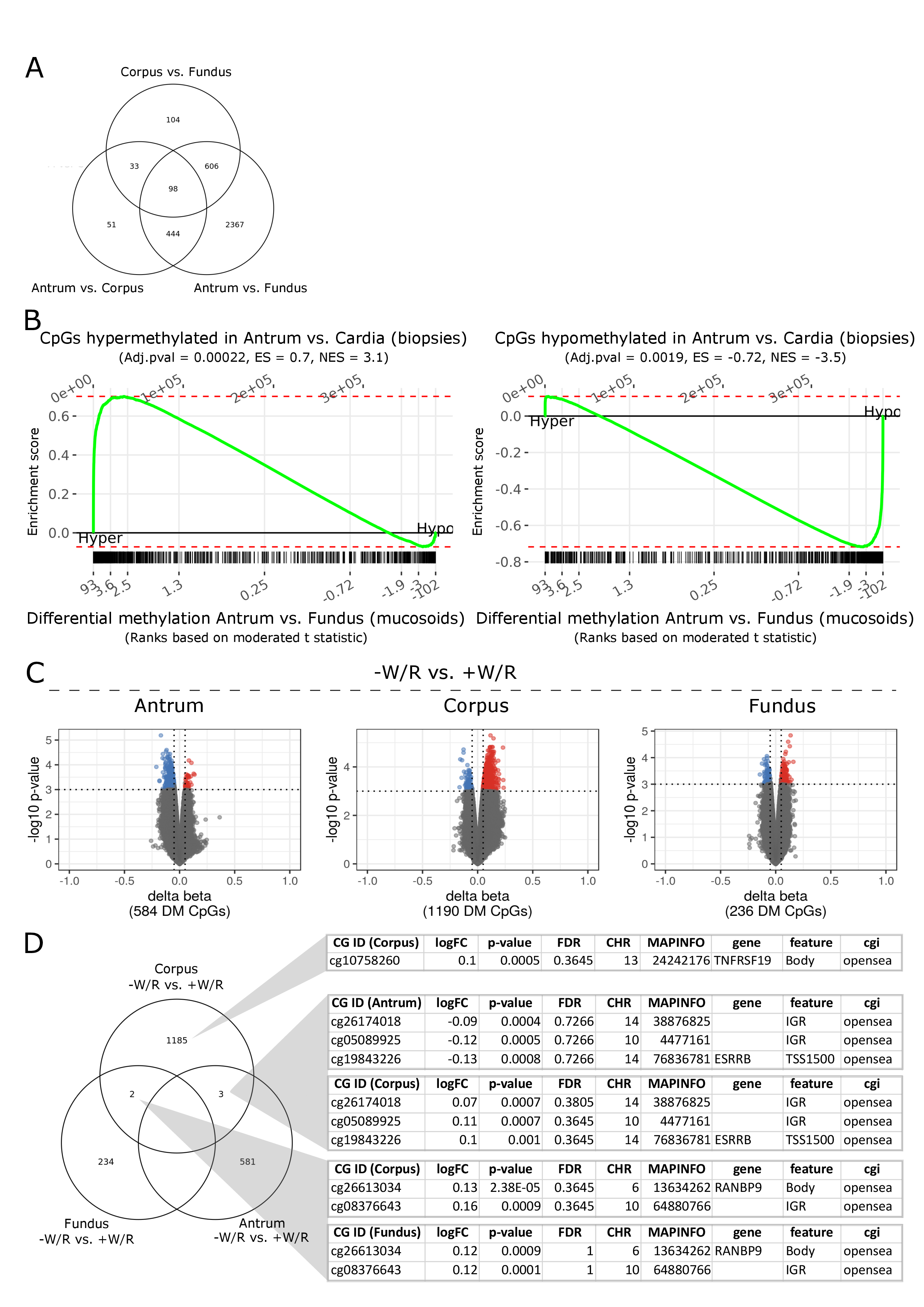

Supplement: Supplementary file 1 — Additional file 1: Figure S1. A) Venn diagram of differentially methylated (DM) CpGs by regional comparisons of the stomach. B) Enrichment of sets of CpGs hypermethylated (left) or hypomethylated (right) between antrum and cardia biopsies compared to differential methylation of CpGs between antrum and fundus mucosoids. Moderated t-scores were used for DM CpG ranking. ES—enrichment score; NES—normalized enrichment score. Hyper, Hypo indicate the direction of differential methylation in the antrum vs. fundus mucosoids comparison. C) Volcano plots representing comparisons between differentiated (− W/R) vs. undifferentiated (+ W/R) states in each stomach region with a less stringent threshold. Delta beta is plotted against the negative logarithm of the raw p-values. The dashed horizontal and vertical lines indicate cutoffs of p < 0.001 and delta beta between − 0.05 and 0.05, respectively. Red dots refer to hypermethylated CpGs and blue dots to hypomethylated CpGs. D) Venn diagrams showing overlaps of DM CpGs upon differentiation in the antrum, corpus and fundus. The tables provide further details on the overlapping DM CpGs, including stem cell-specific genes (TNFRSF19 = Troy). [file 13148_2022_1406_MOESM1_ESM.tif]

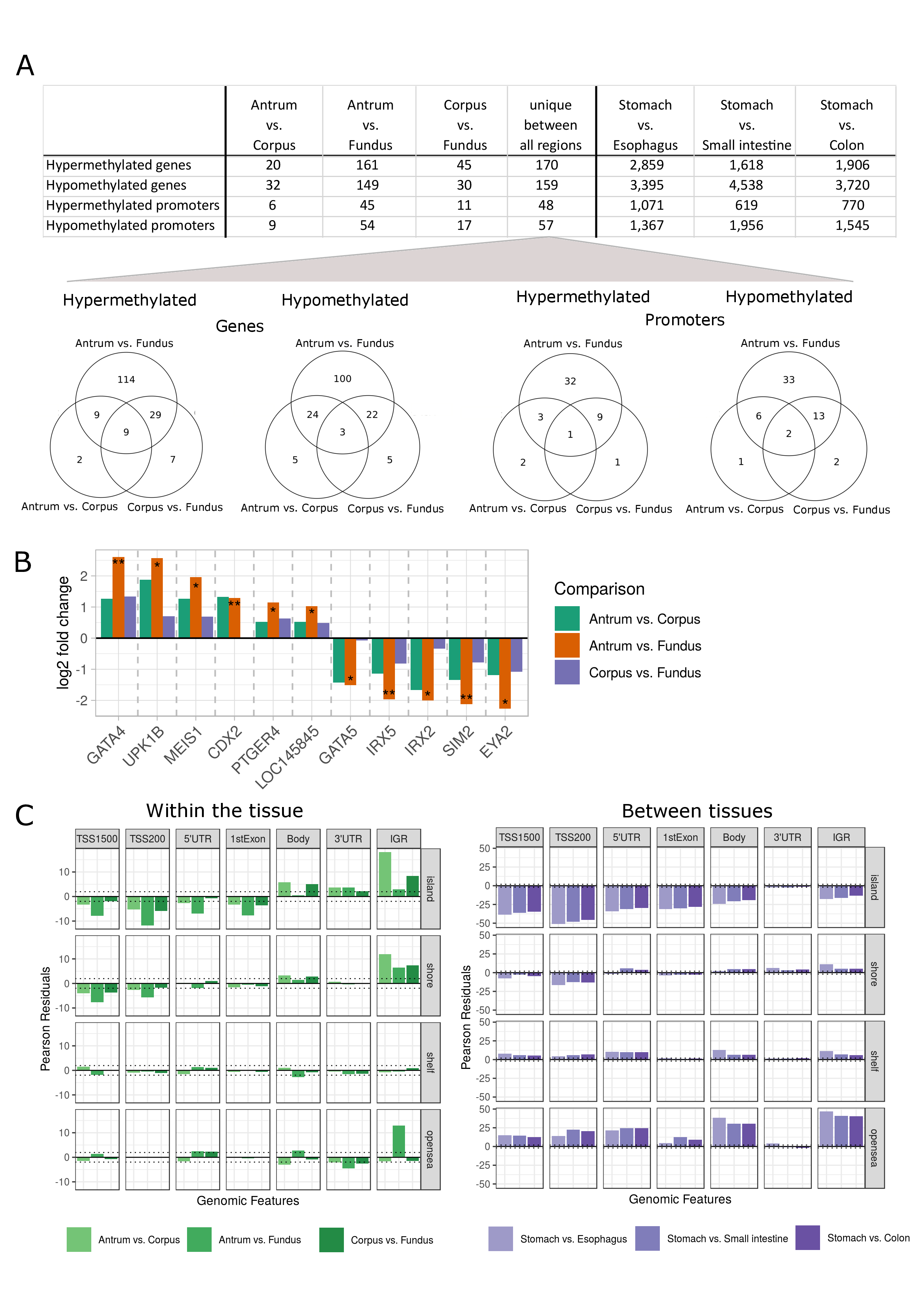

Supplement: Supplementary file 2 — Additional file 2: Figure S2. A) Table of the numbers of hypermethylated and hypomethylated genes and promoters, comparing regions within the stomach and the stomach to adjacent tissues. Bottom: Venn diagrams showing overlaps of DM genes and promoters between the stomach regions. B) Differential gene expression of DM genes between the stomach regions. Shown are genes with log2 fold changes > 0.6 or < − 0.6, * p < 0.01, ** FDR < 0.05. C) DM affects genomic features: comparisons of mucosoid samples from different stomach regions (left) and between the stomach to adjacent tissues (right). The plots show the Pearson residuals of the Chi-square statistic of counts of significant DM CpGs located in in TSS1500, TSS200, 5’UTR, 1stExon, gene body, 3’UTR and IGRs with respect to CpGs islands, shores, shelves and opensea. The dashed line marks the threshold of 95% confidence interval. TSS1500 and TSS200: 200-1500, and 0-200 bases upstream of the transcription start site (TSS); 5’UTR—5’ untranslated region; Body—gene body; 1stExon—first exon; 3’UTR—3’ untranslated region; IGR—intergenomic region. [file 13148_2022_1406_MOESM2_ESM.tif]

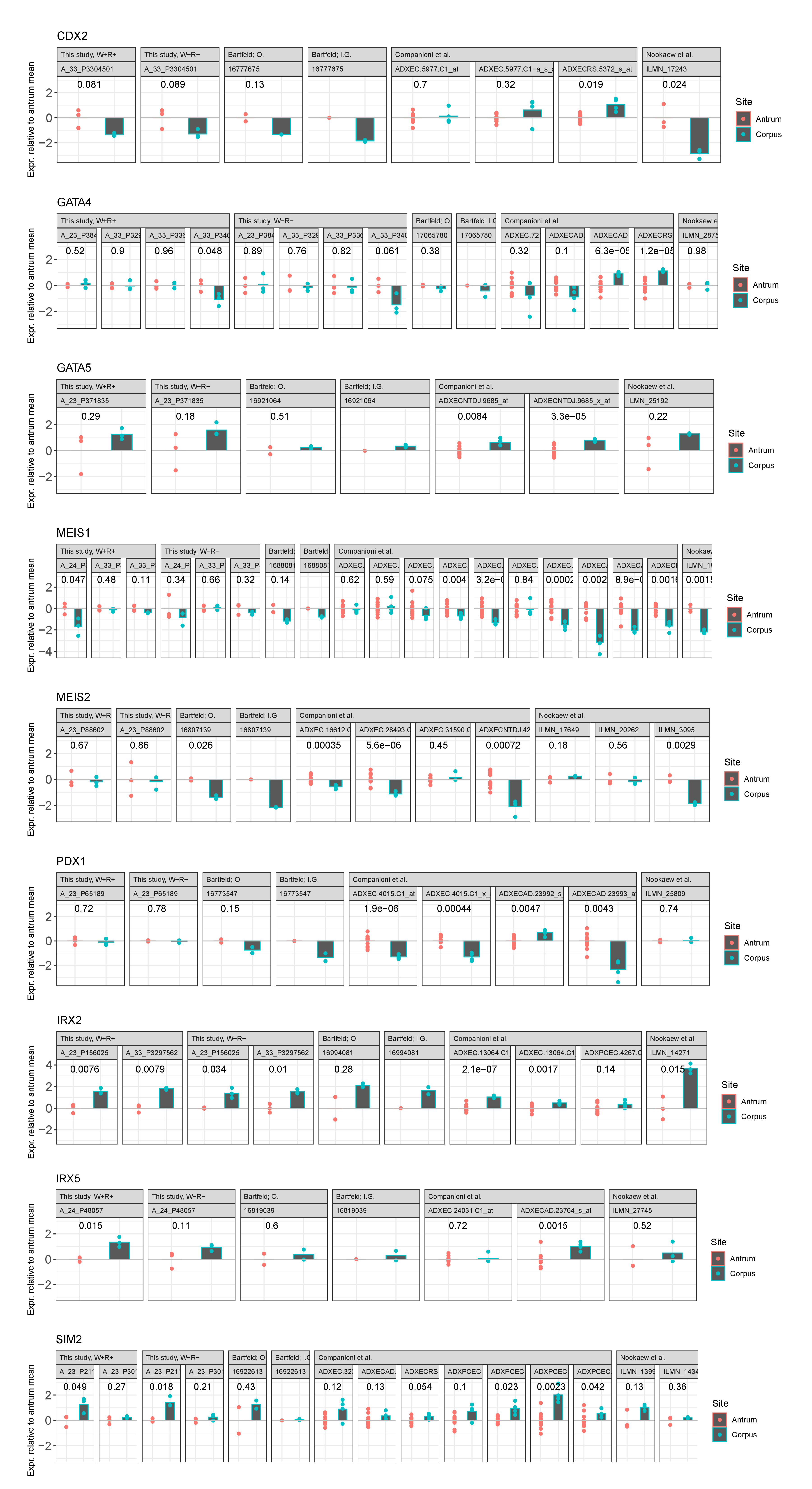

Supplement: Supplementary file 3 — Additional file 3: Figure S3. Differential expression of DM TFs between the antrum and the corpus, based on several in vitro and in vivo data sets (Additional table 7). The expression level is relative to the antrum mean; the p-values of a student t-test between antrum and corpus are shown on each plot, except for cases with less than two samples in each group. For each study, all microarray probes/probe sets mapping to the respective gene are shown. [file 13148_2022_1406_MOESM3_ESM.tif]

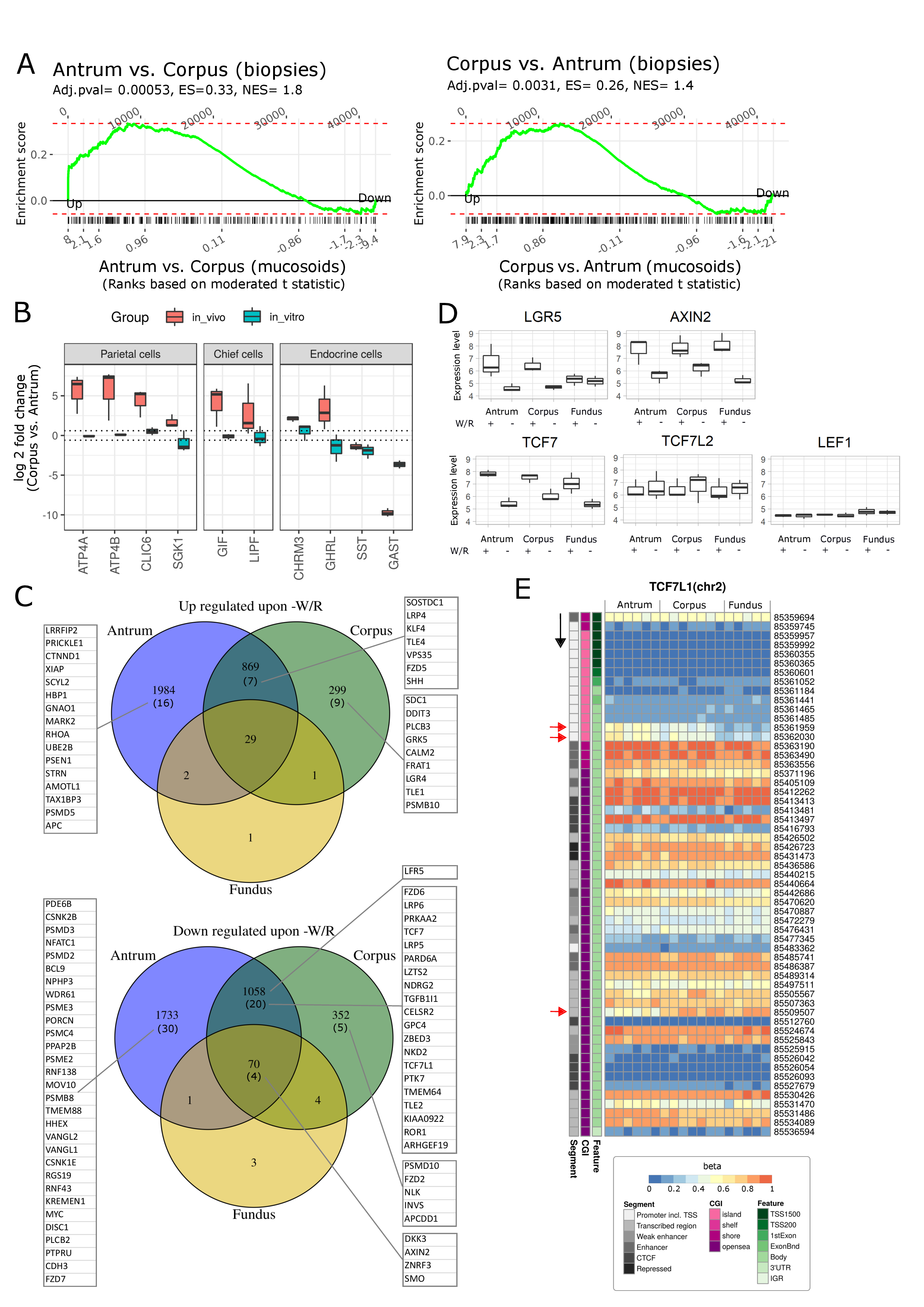

Supplement: Supplementary file 4 — Additional file 4: Figure S4. A) Gene set enrichment analysis of genes overexpressed in antrum (left) or corpus (right) biopsies compared to differential gene expression between antrum and corpus mucosoids. + W/R and − W/R samples of each region were combined for the mucosoid analysis. Moderated t-scores were used for differential expression ranking. Gene sets of biopsies were determined from (Nookaew et al., 2013, Additional table 7). ES—enrichment score; NES—normalized enrichment score. B) Boxplots of biopsy (red) and mucosoid (green) log2 gene expression fold changes, comparing the corpus and the antrum of selected cell-type-specific genes. The dashed line marks the threshold of log2 fold change > 0.6 or < − 0.6. Biopsy samples were taken from Nookaew et al., 2013 (Additional table 7). C) Venn diagrams showing the overlaps of significant upregulated (top) and downregulated (bottom) genes, comparing -W/R (differentiated) to + W/R (undifferentiated) in the different stomach regions (FDR < 0.2; log2 fold change > 0.6 or < − 0.6). Numbers in brackets indicate the number of genes belonging to the GO Wnt signaling pathway gene set, and the corresponding genes are listed accordingly. D) Normalized expression boxplots of Wnt signaling pathway genes. The boxplots display the median, minimum and maximum normalized expression values of three biological replicates. E) Heatmap of TCF7L1 DNA methylation. Displayed are the normalized methylation values (beta) ranging from 0 (blue, unmethylated) to 1 (red, methylated). Significantly DM CpGs between the antrum and the fundus are marked with red arrows. The black arrow refers to the transcriptional direction. CGI—CpG island, TSS—transcription start site, 5’UTR—5’ untranslated region, IGR—intergenomic region. [file 13148_2022_1406_MOESM4_ESM.tif]

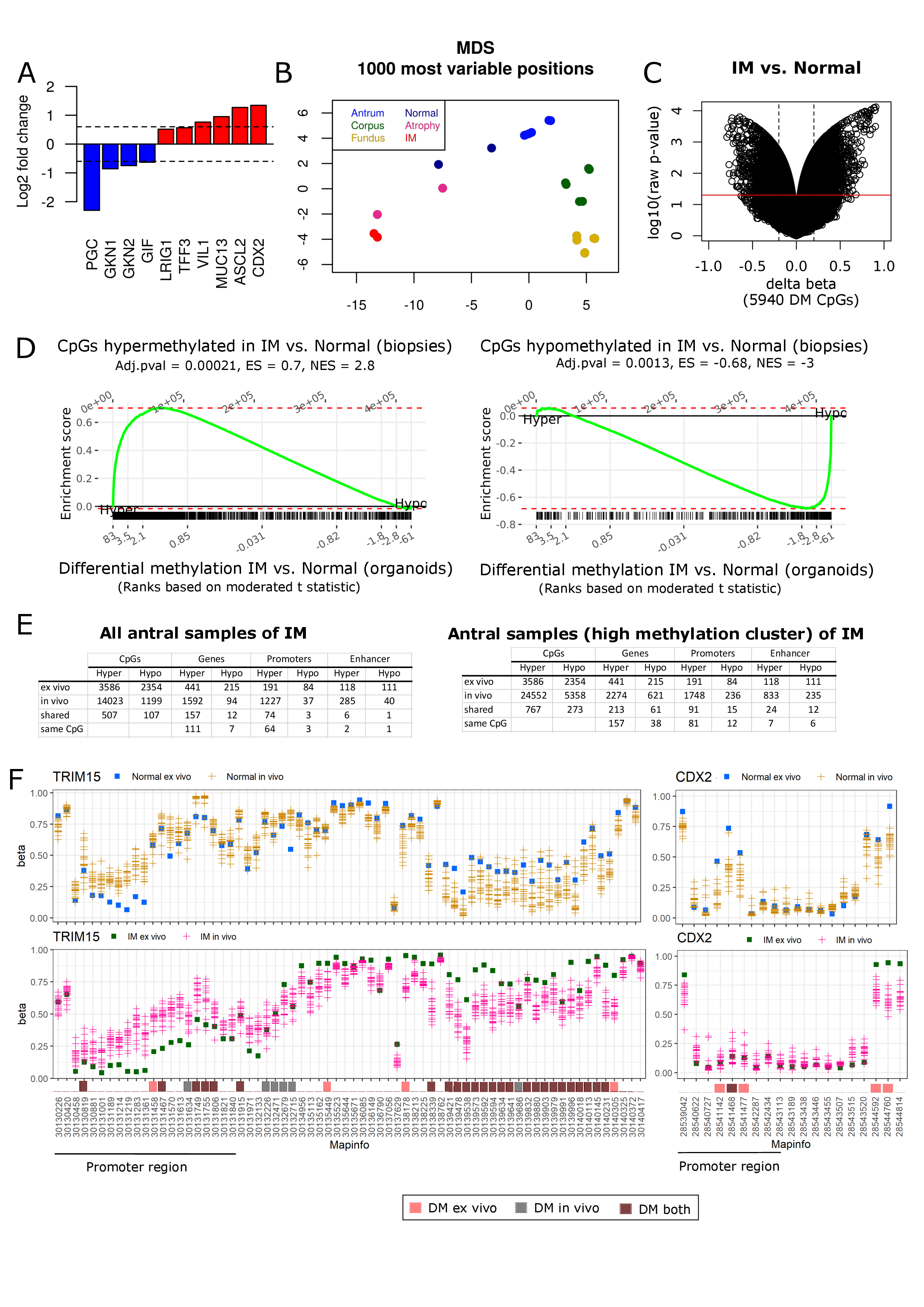

Supplement: Supplementary file 5 — Additional file 5: Figure S5. A) Gene expression log2 fold changes of the stomach- and intestine-specific genes comparing IM and normal organoids (FDR < 0.05). The dashed line indicates a cutoff of log2 fold change > 0.6 or < − 0.6. B) DNA methylation MDS plot of healthy mucosoids from the antrum, corpus and fundus, or normal, atrophic and intestinal metaplasia (IM) organoids. C) Volcano plot of IM vs. normal organoids. Changes in methylation (delta beta) are plotted against the negative logarithm of p-values. The red horizontal and the dashed vertical lines indicate cutoffs of p < 0.05 and delta beta between -0.2 and 0.2, respectively. D) Enrichment of sets of CpGs hypermethylated (left) or hypomethylated (right) between IM biopsies and normal biopsies compared to differentially methylation of CpGs between IM and normal organoids. Moderated t-scores were used for ranking the CpGs. Hyper, Hypo indicate the direction of differential methylation in the IM vs. normal organoid comparison. E) Number of DM CpGs, genes, promoters and enhancers determined in the comparison of IM samples to healthy samples. Left: organoid and biopsy antral IM samples, right: the corresponding samples of the high methylation clusters. F) Dot plots of methylation levels (beta values) of the genes TRIM15 and CDX2: healthy organoid and biopsy samples (top) and IM organoid and biopsy samples (bottom). Squares and crosses denote organoid and biopsy data, respectively. Colors below the plot show DM calls of IM vs. healthy in both data sets. [file 13148_2022_1406_MOESM5_ESM.tif]

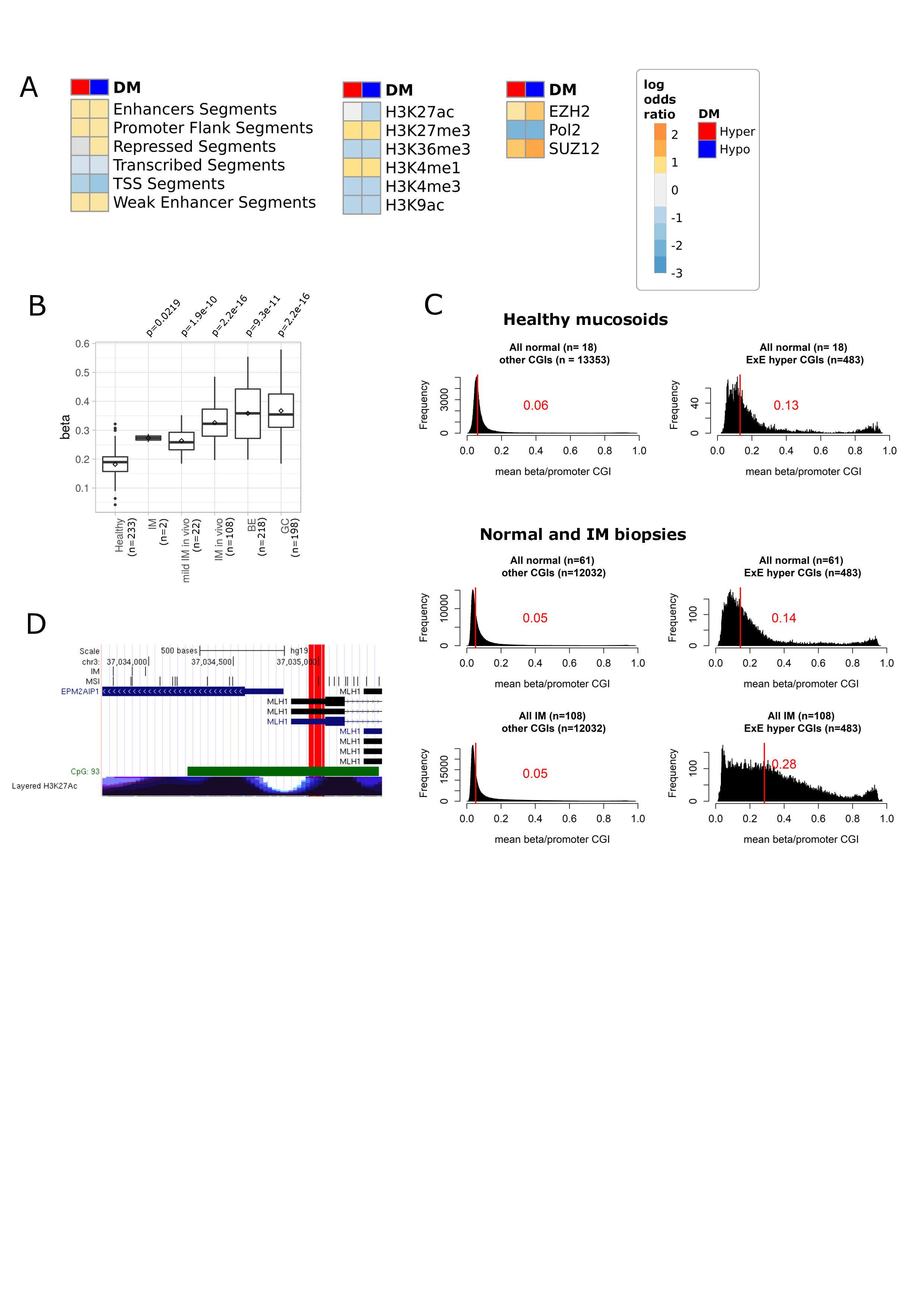

Supplement: Supplementary file 6 — Additional file 6: Figure S6. A) Comparison of organoid IM samples to normal organoid samples—enrichment or depletion of hypermethylated and hypomethylated CpGs in various genomic features. Genomic features were defined based on Encode segmentation classes and transcription factor binding sites (Siggens and Ekwall, 2014), and Roadmap histone modification in gastric tissues (Roadmap Epigenomics Consortium et al., 2015). Shown are log odds ratios of significantly (FDR < 0.05) enriched (log odds ratio > 0.6) or depleted (log odds ratio < − 0.6) DM CpGs. The color code ranges from blue (depleted) to red (enriched), gray refers to a log odds ratio < 0.6 or > − 0.6. Pol2—Polymerase 2 subunit; Hyper—hypermethylated CpGs; Hypo—hypomethylated CpGs. B) Boxplots of ExE CpG island (CGI) hypermethylation. Shown are the mean ExE CGI hypermethylation values per sample. Healthy samples include, in addition to those of the respective data sets, Roadmap epigenome healthy samples (n = 77), except for the extra-embryonic trophoblast cell line and placenta samples. Diseased samples include the respective mild IM and IM (biopsies), IM (organoids), and BE and GC (both biopsies). P values displayed refer to the Wilcoxon rank-sum test with continuity correction. C) Histograms of the methylation distribution in ExE hypermethylated CGI and all other CGI taken from the healthy mucosoid data set of the stomach regions and the data set of normal and IM samples from biopsies. The red line and number indicate the median methylation value. D) UCSC Genome Browser Image of the MLH1 promoter region with custom tracks showing the hypermethylation sites in samples of ex vivo IM and MSI type of GC (Kent et al., 2002). The sites responsible for MLH1 silencing in cancer (Morak et al., 2008) are marked in red. Additional tracks show UCSC CpG islands (green) and the UCSC layered H3K27Ac marks. [file 13148_2022_1406_MOESM6_ESM.tif]
