# Supplementary material for: DNA methylation in human gastric epithelial cells defines regional identity without restricting lineage plasticity
Source: Clin Epigenetics. 2022 Dec 30;14:193. doi: 10.1186/s13148-022-01406-4 (PMC9801550; doi:10.1186/s13148-022-01406-4)
Supplement: Supplementary file 13 — Additional file 13: DNA Methylation and gene expression data sets used in this manuscript. [file 13148_2022_1406_MOESM13_ESM.docx]

**Supplemental Table 7. Data sets created and used in this manuscript**

| Name of the data set | Content | description | GEO accession/  reference |
| --- | --- | --- | --- |
| This study (Mucosoid/ in vitro stomach samples) | DNA methylation and gene expression of healthy gastric epithelial cells | 18 samples  3 biological replicates of   - antrum +W/R and ‑W/R - corpus +W/R and ‑W/R - fundus +W/R and ‑W/R | GSE141660 |
| Healthy stomach in vivo | DNA methylation | 61 normal biopsies   - 39 antrum - 11 corpus - 11 cardia | A subset of GSE103186;  (13) |
|  | Gene expression | 6 normal biopsies (*H. pylori* negative)   - 3 antrum - 3 corpus | A subset of GSE27411 (93) |
|  |  | 15 normal biopsies   - 11 antrum - 4 corpus | A subset of GSE78523 (94) |
| Healthy stomach in vitro | Gene expression | 3 samples of isolated glands   - 1 antrum - 2 corpus   4 samples of organoids   - 2 antrum - 2 corpus | A subset of GSE60557 (95) |
| Combined healthy tissue data set | DNA methylation | 11 normal esophagus biopsies (non-cancer patients) | A subset of GSE72872 (96) |
|  |  | 2 technical replicates of normal small intestine biopsies | GSE67484;  Unpublished; (Habano 2015) |
|  |  | 3 samples of normal colon biopsies | GSE81211;  (56) |
|  |  | 18 mucosoid samples (see above) | GSE141660 |
| This study (normal ex vivo, atrophy, and IM) | DNA methylation and gene expression | 6 samples  2 biological replicates of   - Normal - Atrophic - IM | GSE141660 (DNA methylation and gene expression) |
| Intestinal metaplasia (IM) in vivo | DNA methylation | 191 samples   - 39 normal antrum - 76 IM antrum - 11 normal corpus - 11 normal cardia - 22 mild IM antrum - 23 IM corpus - 9 IM cardia | A subset of GSE103186;  (13) |
| Antrum in vivo | DNA methylation | 8 samples of antral biopsy (*H. pylori* negative) | A subset of GSE92863 (97) |
| Corpus in vivo | DNA methylation | 14 samples of corpus biopsy (*H. pylori* negative) | A subset of GSE99553 (98) |
| Barretts’s esophagus (BE) | DNA methylation | 83 samples   - 64 normal esophagus (BE- and cancer patients) - 11 normal esophagus (non-cancer patients) - 19 BE | A subset of GSE72872;  (56) |
| Combined healthy samples *in vitro*, IM *ex vivo*, and TCGA STAD data set | DNA methylation | 244 samples   - 18 mucosoid samples - 6 ex vivo samples - 220 TCGA GC samples   - 45 MSI (22 antrum, 20 corpus, 3 GECA)   - 48 GS (24 antrum, 17 corpus, 7 GECA)   - 105 CIN (38 antrum, 44 corpus, 23 GECA) | A subset of the TCGA STAD data set (excluding EBV) (14) |
| GTEx | Gene expression of STAD | 139 normal stomach samples in comparison with TCGA STAD | (76) |
| Human Protein Atlas | Stomach specific genes | 166 genes with five-fold higher mRNA levels compared to averaged levels in all other tissues | (50) |
| Chief and parietal GE | Chief and parietal cell signature | Mice chief and parietal cells obtained from laser Capture Microdissection | A subset of GSE5018 (83) |
| Troy^+^ cells GE | Troy+ cell signature | Significantly enriched genes in Troy+ chief cells compared to whole corpus gland (mice) | Supplementary Table1 (45) |
| Beta Catenin target genes | Beta Catenin target genes | β-catenin target genes in human colon or colon carcinoma cells (including colon carcinoma cell lines) | Table 1 (90) |

W/R = Wnt and R-spondin; IM = Intestinal metaplasia; BE = Barrett’s esophagus; STAD = stomach adenocarcinoma.

References in the table refer to the bibliography in the main text. Additional references not listed in the main text:

93. Nookaew I, Thorell K, Worah K, Wang S, Hibberd ML, Sjövall H, et al. Transcriptome signatures in Helicobacter pylori-infected mucosa identifies acidic mammalian chitinase loss as a corpus atrophy marker. BMC Med Genomics. 2013;11(6):41.

94. Companioni O, Sanz-Anquela JM, Pardo ML, Puigdecanet E, Nonell L, García N, et al. Gene expression study and pathway analysis of histological subtypes of intestinal metaplasia that progress to gastric cancer. PLoS ONE. 2017;12(4):e0176043.

95. Bartfeld S, Bayram T, van de Wetering M, Huch M, Begthel H, Kujala P, et al. In vitro expansion of human gastric epithelial stem cells and their responses to bacterial infection. Gastroenterology. 2015;148(1):126-136.e6.

96. Kang K, Bae JH, Han K, Kim ES, Kim TO, Yi JM. A genome-wide methylation approach identifies a new hypermethylated gene panel in ulcerative colitis. Int J Mol Sci. 2016;17(8):E1291.

97. Yamashita S, Nanjo S, Rehnberg E, Iida N, Takeshima H, Ando T, et al. Distinct DNA methylation targets by aging and chronic inflammation: a pilot study using gastric mucosa infected with Helicobacter pylori. Clin Epigenet. 2019;11(1):191.

98. Woo HD, Fernandez-Jimenez N, Ghantous A, Degli Esposti D, Cuenin C, Cahais V, et al. Genomewide profiling of normal gastric mucosa identifies Helicobacter pylori- and cancer-associated DNA methylome changes. Int J Cancer. 2018;143(3):597–609.
